# The antibacterial effect of whey protein–alginate coating incorporated with the lactoperoxidase system on chicken thigh meat

**DOI:** 10.1002/fsn3.634

**Published:** 2018-03-23

**Authors:** Roghayeh Molayi, Ali Ehsani, Mohammad Yousefi

**Affiliations:** ^1^ Department of Nutrition Faculty of Nutrition and Food Science Tabriz University of Medical Sciences Tabriz Iran; ^2^ Department of Food Science and Technology Faculty of Nutrition and Food Science Tabriz University of Medical Sciences Tabriz Iran; ^3^ Department of Food Science and Technology Faculty of Nutrition and Food Science Talented Students Center Student Research Committee Tabriz University of Medical Sciences Tabriz Iran

**Keywords:** chicken thigh, coating, lactoperoxidase, whey protein–alginate

## Abstract

Nowadays, the environmental problems due to the use of synthetic films and packages have caused the production of natural edible coatings or films. The aim of this study was to produce an edible whey protein–alginate coating with different concentrations of lactoperoxidase system to control the microbial load and increase the shelf life of chicken thigh meat stored in refrigerated condition (4 ± 1°C). So, after the provision of the alginate–whey protein coating incorporated with the lactoperoxidase system (at concentrations of 2%, 4%, and 6% in alginate–whey protein solution), microbial experiments were conducted for the period of 8 days. Three batches of organisms, including total aerobic mesophilic bacteria, *Enterobacteriaceae,* and *Pseudomonas aeruginosa* in samples, were tested by culturing in appropriate conditions. Results indicated that the coating had a substantial inhibitory effect on all lots. Also, the antimicrobial activity of coating increased with increase in lactoperoxidase system concentration in alginate–whey protein coating.

## 1 INTRODUCTION

Henson and Traill defined the food safety as the inverse of food risk—the probability of not suffering some hazard from consuming a specific food (Henson & Traill, 1993). Food is a critical contributor to physical well‐being and a major source of pleasure, worry, and stress (Lake et al., 2012). Each food item must be safe, aesthetically pleasing, and have good taste. According to the Center for Disease Control and Prevention, an estimated 20,000 Americans are poisoned by this particular strain of *Escherichia coli* annually and, of these, about 250 persons die (Wilcock, Pun, Khanona, & Aung, 2004). Although the incidence of bacterial contamination of foods is occasional, their consequences are vast, resulting in illnesses, deaths, and loss of production and public confidence in the food industry (Painter et al., 2013).

Edible coatings can increase the quality and shelf life of foods by reducing the penetration of water, the degree of shrinkage, and the microbial contamination, likewise by keeping the taste and postponing the fat oxidation. These coatings are formed from protein, carbohydrate, or lipid substances. Alginate and whey protein as the subsets of carbohydrate and protein materials are of the most applicable natural coatings used in food industries (Falguera, Quintero, Jiménez, Muñoz, & Ibarz, 2011; Yousefi, Azizi, Mohammadifar, & Ehsani, 2018).

Alginate is a salt of alginic acid, a polymer of d‐mannuronic acid and l‐guluronic acid, and it is isolated from brown algae (Chidanandaiah, Keshri, & Sanyal, 2009). Alginate has unique colloidal properties and can form strong gels or insoluble polymers through cross‐linking with calcium. Such biopolymer‐based films can keep the good quality and prolong the shelf life of foods. Alginate likewise has been investigated as an effective active coating against *Listeria monocytogenes* on poached and deli turkey products (Juck, Neetoo, & Chen, 2010). Also, alginate is a GRAS (generally recognized as safe) substance (Dettmar, Strugala, & Richardson, 2011).

Since the early 90s, researchers found that the whey protein has the ability to form film as a natural polymer composition and can be used as an alternative to synthetic polymers (Murray, 2011). Whey protein‐based coatings in addition to improving the nutritional value, have good mechanical properties, produce transparent films or coatings, and have better permeability than the films prepared from carbohydrates and fats (Kaplan, Wardowski, Nagy, & Grierson, 1986; Min, Harris, & Krochta, 2005).

Lactoperoxidase system (LPOS) is a natural antimicrobial system in milk and in human secretions such as saliva and tears (Kussendrager & van Hooijdonk, 2000). The use of LPOS has been suggested as a preservative in foods and pharmaceuticals (Bosch, Van Doorne, & De Vries, 2000). The example is the application of whey protein coating incorporated with LPOS in Pike‐Perch fillets (Shokri & Ehsani, 2017).

Chicken thigh meat is one of the most important sources providing the human body protein. Also, poultry meats have high consumption due to their low cost compared to red meats. Therefore, this study was carried out to found the effect of alginate–whey protein coating in controlling the bacterial contaminating of chicken thigh meat stored in the refrigerator (4°C).

## 2 MATERIALS AND METHODS

### 2.1 Materials

Materials, including whey protein isolate (WPI), lactoperoxidase enzyme (150 U/mg, Sigma‐Aldrich), α‐d‐glucose (Sigma‐Aldrich), glucose oxidase (Sigma‐Aldrich), H_2_O_2_ (Merck, Germany), potassium thiocyanate (Bio Serae, France), carboxymethyl cellulose (Sigma‐Aldrich), alginic acid (Sigma‐Aldrich), calcium chloride (Sigma‐Aldrich), and glycerol (Frankfurt, Germany), were purchased.

### 2.2 Raw material

Boneless, skinless chicken thigh meats were purchased from the butcher shops. Samples were prepared in an average weight of 10 ± 0.5 g. All samples, including control and treated, were packed in polypropylene bags and kept under refrigerated condition (4 ± 1°C) for 8 days.

### 2.3 Preparation of LPOS

The LPOS was composed of LPO, glucose oxidase (GO), α‐d‐glucose (Glu), potassium thiocyanate (KSCN), and hydrogen peroxide (H_2_O_2_.). The ratio of components used in the system was 1.00:0.35:108.70:1.09:2.17 in the order of LPO, GO, Glu, KSCN, and H_2_O_2_. The selected concentrations were according to other studies (Atamer et al., 1999). The ingredients were dissolved in 50 ml of 50 mmol/L phosphate buffer (pH 7.4). The solution was incubated at 23 ± 2°C for 24 hr using a water bath shaker (with shaking at 160 revolutions per minute) to intensify the antimicrobial activity of LPOS (Bosch et al., 2000).

### 2.4 Whey protein coating preparation

The whey protein coating was prepared as described by Han and Krochta (2007). Whey protein isolate (WPI) powder was dissolved in deionized water at 10% (w/w) concentration. Glycerol (Gly) was added to 10% WPI solution at the ratio of 2%. The solution was heated for 30 min in a 90°C circulatory water bath for denaturation of WPI and forming gel and then was cooled in an ice bath.

### 2.5 Alginate coating preparation

The alginate coating was prepared as described by Song, Liu, Shen, You, and Luo (2011) with a little modification. In order to avoid the formation of calcium alginate gel before application on samples, two solutions were used to prepare the coating solution.

Solution 1: Thirty grams of alginate with 1000 ml of distilled water was mixed and stirred at a controlled temperature of 80°C until the mixture became clear. Then, 20 ml glycerin was mixed with the prepared sodium alginate solution and stirred thoroughly. Then, the well‐mixed solution was made up to 2000 ml with distilled water.
Solution 2: Two percent (w/v) calcium chloride was also prepared.

### 2.6 Alginate–whey protein with LPOS coating preparation

After preparing the coatings, LPOS was added at concentration levels of 2, 4, 6, and 8% (v/v) to the second solution and agitated vigorously.

### 2.7 Coating of chicken thigh meats

Samples in the weight of 10 ± 0.5 g were immersed in whey protein solution for 60 seconds, and then samples were allowed to drip extra solution for 30 s. After that, samples immersed in solution 1 for 3 s, and then after dripping the extra solution drops, samples subsequently were soaked in solution 2 for 30 s. All samples were preserved in polyethylene bags under refrigerated condition for 8 days.

### 2.8 Antibacterial activity of coatings

The disk diffusion method with the purpose of finding the antibacterial activity of coatings with different LPOS concentrations was performed. For this purpose, two strains of bacteria, including *Pseudomonas fluorescens* NCTC 10038 and *E. coli NCTC 12241*, were used.

### 2.9 Disk diffusion method

Disk diffusion method was carried out as described by Kirby‐Bauer (K‐B) (Piozzi, Francolini, Occhiaperti, Venditti, & Marconi, 2004).

LPOS at concentrations of 0.5%, 1%, 2%, 4%, 6%, 8%, and 10% was added to second solution. Bacterial suspensions (*Pseudomonas fluorescens* and *E. coli*) were adjusted to 1.5 × 10^8^ CFU/ml by 0.5 McFarland solution and then were spread on the surface of Muller Hinton agar using sterile cotton swabs. Afterward, small filter blank disks (6 mm diameters) were impregnated with coating solution and placed on the surface of the medium. The plates were incubated at 37°C for 24–48 hr. The strength of antibacterial activity of coatings was estimated by observing the diameter of the inhibition zone. Also, the different ratios of alginate to whey protein, including 0%, 25%, 50%, 75%, and 100% alginate solution, were tested to found the best ratio in coating to control bacterial growth.

### 2.10 Coating formulation

Samples were arranged into five coating formulations as follows:

1. Coating formulation: whey protein–alginate coating with no LPOS (C‐0)
2. Coating formulation: whey protein–alginate coating with 2% LPOS (C‐2)
3. Coating formulation: whey protein–alginate coating with 4% LPOS (C‐4)
4. Coating formulation: whey protein–alginate coating with 6% LPOS (C‐6)
5. Coating formulation: whey protein–alginate coating with 8% LPOS (C‐8)

### 2.11 Evolution of the microbial spoilage

The effect of whey protein–alginate–LPOS coating on the microbial development in chicken thigh meat samples preserved under refrigerated condition on days 0, 2, 4, 6, and 8 was evaluated. Microbiological analyses were focused on the following: total aerobic mesophilic bacteria (AMB), *Enterobacteriaceae*, and *Pseudomonas aeruginosa*. For this purpose, 10 g of samples were put in a sterile plastic bag with 90 ml of 0.1% peptone water and homogenized in a stomacher for 1 min. Appropriate decimal dilutions were prepared and inoculated over plate count agar (PCA), violet red bile agar (VRBA), and cetrimide agar mediums for culturing AMB, *Enterobacteriaceae*, and *Pseudomonas aeruginosa*, respectively. Plates were incubated at 37°C for 24–48 hr.

### 2.12 Statistical analysis

All tests were performed in duplicate. All data were subjected to the analysis of variance (ANOVA) and Duncan test using SPSS version 16. Significance was accepted at *p* < .05.

## 3 RESULTS AND DISCUSSION

### 3.1 Disk diffusion test

It was observed that the ≤1% LPOS concentration produced none or small growth inhibition zone, while the concentration of ≥2% showed large inhibition zone. Also, the concentration of 10% produced a viscous and dark yellow solution. Therefore, the levels of 2%, 4%, 6%, and 8% were selected.

Also, it was found that 50–50 proportion of whey protein–alginate solution produced large growth inhibition zone. Therefore, the 50–50 proportion of whey protein–alginate was selected to be used as coating. The results of disk diffusion test are shown in Table 1.

**Table 1:** The results of disk diffusion of different levels of lactoperoxidase system in the coating solution

Bacteria	Volume of the coating solution on blank disks (ul)	Concentration of LPOS (%) in 50:50 proportion of whey protein–alginate	Inhibition zone (mm)
*Pseudomonas fluorescens*	20	0.5	6
	20	1	8
	20	2	10
	20	4	14
	20	6	17
	20	8	22
	20	10	25
*Escherichia coli*	20	0.5	6
	20	1	7
	20	2	12
	20	4	13
	20	6	16
	20	8	20
	20	10	26

### 3.2 *Enterobacteriaceae* changes

**Figure 1:**
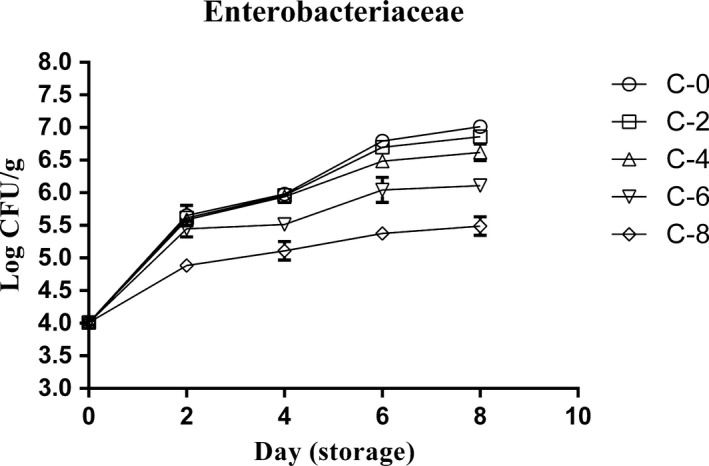
*Enterobacteriaceae* changes of chicken thigh meat as affected by whey protein–alginate coating incorporated with the LPOS during refrigerated storage (4 ± 1°C). Values are the mean ± *SD*

*Enterobacteriaceae* counts were ranged from 4.01 to 7.02 logs CFU/g during 8 days. The results (Figure 1) indicated that bacterial growth reduced with increasing the concentration of LPOS in all samples, so that the number of bacteria in C‐8 samples was significantly less (*p* < .05) than other groups on all days except day 0. Also, after the day 2, C‐6 samples demonstrated lower *Enterobacteriaceae* counts compared to C‐0, C‐2, and C‐4 samples. But, the findings did not show any significant differences (*p* > .05) between C‐0, C‐2, and C‐4 batches on days 0, 2, and 4. On the other hand, the differences between C‐0, C‐2, and C‐4 groups on days 6 and 8 were low and probably meaningless. Concerning the effect of storage day, the only significant difference (more than 1 log CFU/g) was found among all samples, except C‐8 group between days 0 and 2. At other days, the diagram of all samples increased with a slight gradient. Although, with increasing the LPOS concentration, the intensity of the growth of the bacteria became milder.

The *Enterobacteriaceae* family is used commonly as an indicator of fecal contaminations. They include important zoonotic bacteria such as *Salmonella* spp.*, Yersinia* spp., and *E. coli*. *Enterobacteriaceae* are the significant causes of serious infections, and many of them are becoming resistant to currently available antimicrobials (Paterson, 2006). As it was conceived before, lactoperoxidase as a gram‐negative antibacterial agent impacted on *Enterobacteriaceae* growth, especially at high concentration in coatings. According to the results (Figure 1), two levels of 6 and 8% LPOS significantly reduced the *Enterobacteriaceae* counts during 16 days of storage.

Concerning the studies conducted about the effect of LPOS coatings on *Enterobacteriaceae*, there are limited sources. But, some works have showed similar results to our findings. For example, results of Gurtler et al. (Gurtler & Beuchat, 2007) study about the controlling *Enterobacter sakazakii* counts in reconstituted powdered infant formula by lactoperoxidase system are in agreement with those found in our study. Gurtler et al. indicated that a dose of 10 μg/ml of lactoperoxidase system significantly decreased *Enterobacter sakazakii* in samples stored at 21, 30, or 37°C. In another work, Yener, Korel, and Yemenicioglu (2009) investigated the effect of LPOS incorporated into cross‐linked alginate films on *E. coli, L. innocua*, and *P. fluorescens*. They tested the impact of different doses of H_2_O_2_ and KSCN in LPOS on aforementioned bacteria. Their results showed that the presence of 4 mmol/L KSCN and 0.2 mmol/L H_2_O2 had low impact on *E. coli*. However, a significant inhibitory effect on *E. coli* was observed when H_2_O_2_ was increased to 0.4 or 0.8 mmol/L.

Regarding the storage days, our results exhibited that the most impact of LPOS occurs after day 2. This finding shows that LOPS needs at least 2 days to be fully activated in alginate solution against *Enterobacteriaceae*. After day 2, as the concentration of LPOS increased, the slope of bacterial growth was reduced, so that the samples containing the lower amount of LPOS have more differences at bacterial counts between the days 0 and 8. These findings are in agreement with Yousefi et al. who investigated the effect of the alginate–LPOS coatings on bacterial growth in chicken breast fillets. That study demonstrated that the higher level of LPOS has more controlling impact on *Enterobacteriaceae* with the passage of days (Yousefi, Farshidi, & Ehsani, 2018).

### 3.3 *Pseudomonas aeruginosa* changes

**Figure 2:**
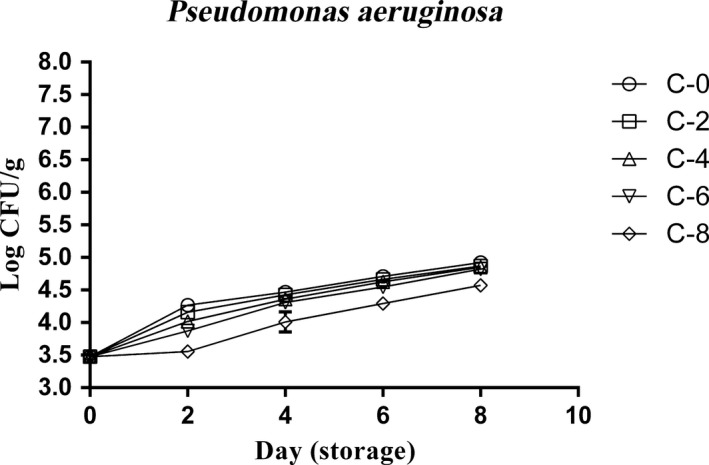
*Pseudomonas aeruginosa* changes of chicken thigh meat as affected by whey protein–alginate coating incorporated with the LPOS during refrigerated storage (4 ± 1°C). Values are the mean ± *SD*

*Pseudomonas aeruginosa* counts were ranged from 3.48 to 4.95 logs CFU/g during 8 days. Therefore, the total growth of *Pseudomonas aeruginosa* was low. ANOVA analysis (Figure 2) indicated that, although there was a significant difference (*p* < .05) between all samples on the second day, but the *Pseudomonas aeruginosa* number in all groups except C‐8 was nearly similar. Also, findings showed that C‐8 samples had lower bacterial (*p* < .05) counts compared to others after day 0.

*Pseudomonas aeruginosa is* the most frequent gram‐negative bacterium associated with nosocomial and life‐threatening chronic infections (Rasheed et al., 2016). Oxygen is vital for such organism due to its absolutely aerobic nature. It has been proved that whey protein has antioxidant property, in addition to antimicrobial and anti‐inflammatory traits (Kishta, Iskandar, Dauletbaev, Kubow, & Lands, 2013). So, it can prevent the growth of absolute aerobic bacteria depending on its concentration in coatings or films. Regarding the relationship between *P. aeruginosa* and edible films, it should be mentioned that *P. aeruginosa* is often used as a model organism for the study of biofilms (Rasheed et al., 2016).

According to our survey, any study about the antimicrobial effect of the whey protein–alginate coating with LPOS on the *Pseudomonas* has not been carried out. However, in a study, it was shown that glucose oxidase (5 U/ml) decreased the number of *P. aeruginosa* cells from 3.03 × 10^5^ CFU/ml to 1.13 × 10^4^ CFU/ml, and the bactericidal activity of glucose oxidase increased by decreasing the pH to 5 or 6 or by combining the glucose oxidase with the lactoperoxidase in biofilms (Johansen, Falholt, & Gram, 1997).

In our study, whey protein–alginate coating incorporated with LPOS can be introduced as a semisuccessful protection agent against *P. aeruginosa*. According to Figure 2, increasing the LPOs concentrations from C‐0 to C‐6 samples slightly decreased the *P. aeruginosa* counts. But, C‐8 sample had the significantly (*p* < .05) lower *P. aeruginosa* in comparison with others. In other hand, it was semisuccessful agent.

### 3.4 Total aerobic mesophilic bacteria (AMB) changes

**Figure 3:**
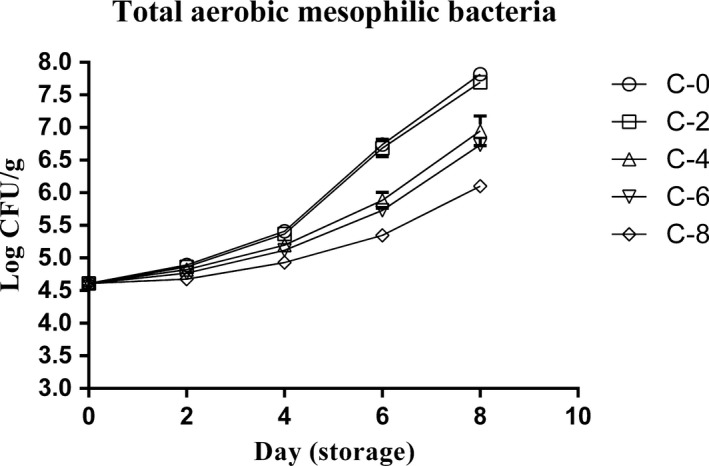
Total aerobic mesophilic bacteria (AMB) changes of chicken thigh meat as affected by whey protein–alginate coating incorporated with the LPOS during refrigerated storage (4 ± 1°C). Values are the mean ± *SD*

AMB counts were ranged from 4.61 to 7.89 logs CFU/g during 8 days. According to the results (Figure 3), differences between all batches were small until day 4. In addition, results did not show any significant difference (*p* > .05) between C‐0 and C‐2 samples on all days. After day 4, C‐4 and C‐6 groups demonstrated significant lower AMB counts (*p* < .05) compared with C‐0 and C‐2 samples, although the significant difference between them (C‐4 and C‐6) was not seen. Also, the findings showed that C‐8 samples had meaningfully lower number of AMB than other groups after day 4.

Regarding the viability of AMB under the protection of antimicrobial coatings, Shokri, Ehsani, and Jasour (2015) displayed that a whey protein–LPOS coating is able to significantly reduce the growth rate of such organisms. Shokri et al. made a whey protein coating incorporated with levels of 0% to 7.5% LPOS and tested the coating effect on a group of bacteria existing in rainbow trout fillets. Their results demonstrated that the antibacterial strength of the coating significantly increased by increasing the concentration of LPOS during 16 days. In our study, likewise, it was showed that adding LPOS in concentrations of more 2% had significantly reducing effect on AMB. Besides, the concentration of 8% LPOS had the most effect on decreasing the growth rate of bacteria.

In other study performed by Chidanandaiah et al. in 2009 (Chidanandaiah et al., 2009), it was exhibited that alginate coating without any antibacterial agents meaningfully reduced the AMB growth rate in meat patties. Although we did not evaluate the bacterial growth in samples without coating, it can be deduced that alginate–whey protein coating probably should have the controlling influence on AMB.

Concerning the simultaneous effect of storage time and LPOS concentration, our research shows that, with increasing the both of them, the differences between the LPOS containing samples with C‐0 group increase significantly. Also, it has been demonstrated that LPOS needs at least 4 days to deal with the AMB growth effectively. These conclusions are compatible with Yousefi et al. who observed that chicken breast fillets containing alginate coating incorporated with 6% LPOS has significant lower AMB counts compared with control samples without LPOS on days 8, 12, and 16. Also, they did not see any differences between samples until the fourth day of storage in refrigerator (Yousefi, Farshidi, et al., 2018).

In other survey, Shokri et al. investigated the efficacy of whey protein coating incorporated with LPOS on bacterial growth in Pike‐Perch fillets during refrigeration. Contrary to our results, Shokri et al. indicated that the 2.5% LPOS in whey protein coating has meaningful impact on total viable bacteria. This result is probably because of differences in the type of sample and coating used in two studies (Shokri & Ehsani, 2017).

## 4 CONCLUSIONS

The whey protein–alginate coating incorporated with the lactoperoxidase system in different levels could significantly control the bacterial growth of tested bacteria, particularly *Enterobacteriaceae* and total aerobic mesophilic bacteria. Also, the study resulted in the increase in antibacterial effect with increasing the LPOS level, so that the most effective coating was related to whey protein–alginate coating with 8% LPOS.

## CONFLICT OF INTEREST

The authors declare that they do not have any conflict of interest.
